# Fluorodeoxyglucose positron emission tomography in the evaluation of germ cell tumours at relapse

**DOI:** 10.1054/bjoc.2000.1389

**Published:** 2000-09-04

**Authors:** S F Hain, M J O’Doherty, A R Timothy, M D Leslie, P G Harper, R A Huddart

**Affiliations:** The Clinical PET Centre; Department of Clinical Oncology, Guy’s and St Thomas’ Hospitals, London; The Royal Marsden Hospital, Surrey, UK

**Keywords:** fluorodeoxyglucose, positron emission tomography, testicular cancer

## Abstract

Differentiation of active disease from fibrosis/mature teratoma in patients with residual masses or identifying of sites of recurrence in patients with raised markers following treatment of their testicular cancer remains a problem.^18^F-fluorodeoxyglucose positron emission tomography (FDG-PET) has the potential to identify active disease and thereby influence further management in these patients. We performed a retrospective study of the use of FDG-PET in detecting residual/recurrent testicular carcinoma in 55 patients (seventy FDG-PET scans). Forty-seven scans were for the assessment of residual masses (18 had raised markers) and 23 scans were for the investigation of raised markers in the presence of normal CT scans. True positive results were based on positive histology or clinical follow-up. FDG-PET had a positive predictive value (PPV) of 96% and a negative predictive value (NPV) of 90% in patients with residual masses. This PPV was equivalent to that of markers (94%) but FDG-PET had the advantage of identifying the site of that recurrence. The NPV was higher than that of markers. In patients with raised markers alone the PPV of FDG-PET was 92% but the NPV was only 50%. However, subsequent FDG-PET imaging was frequently the first imaging modality to identify the site of disease. FDG-PET effected a management change in 57% of cases. FDG-PET scanning detected viable tumour in residual masses and identified sites of disease in suspected recurrence. © 2000 Cancer Research Campaign


British Journal of Cancer (2000) 83(7), 863–869
© 2000 Cancer Research Campaign
doi: 10.1054/ bjoc.2000.1389, available online at http://www.idealibrary.com on IDEAL®

### Fluorodeoxyglucose positron emission tomography in the evaluation of germ cell tumours at relapse

**SF Hain[1], MJ O'Doherty[1], AR Timothy[2], MD Leslie[2], PG Harper[2] and RA Huddart[3]**

[1]The Clinical PET Centre; [2]Department of Clinical Oncology, Guy's and St Thomas' Hospitals, London, [3]The Royal Marsden Hospital, Surrey, UK

**Summary** Differentiation of active disease from fibrosis/mature teratoma in patients with residual masses or identifying of sites of recurrence in patients with raised markers following treatment of their testicular cancer remains a problem. $^{18}$F-fluorodeoxyglucose positron emission tomography (FDG-PET) has the potential to identify active disease and thereby influence further management in these patients. We performed a retrospective study of the use of FDG-PET in detecting residual/recurrent testicular carcinoma in 55 patients (seventy FDG-PET scans). Forty-seven scans were for the assessment of residual masses (18 had raised markers) and 23 scans were for the investigation of raised markers in the presence of normal CT scans. True positive results were based on positive histology or clinical follow-up. FDG-PET had a positive predictive value (PPV) of 96% and a negative predictive value (NPV) of 90% in patients with residual masses. This PPV was equivalent to that of markers (94%) but FDG-PET had the advantage of identifying the site of that recurrence. The NPV was higher than that of markers. In patients with raised markers alone the PPV of FDG-PET was 92% but the NPV was only 50%. However, subsequent FDG-PET imaging was frequently the first imaging modality to identify the site of disease. FDG-PET effected a management change in 57% of cases. FDG-PET scanning detected viable tumour in residual masses and identified sites of disease in suspected recurrence. © 2000 Cancer Research Campaign

**Keywords:** fluorodeoxyglucose; positron emission tomography; testicular cancer

Germ cell tumours (seminoma and nonseminomatous germ cell tumours (NSGCT)) of the testis are relatively uncommon, accounting for only 1% of cancers in men, however they are the commonest tumours in young men (15–35 y) and the incidence is rising (Mead, 1995). Although the treatment pathways differ in both groups the overall prognosis is good. Differences in therapy are related to the biological behaviour and metastatic potential of the different tumour types.

Patients with metastatic disease frequently have residual masses following treatment and the management of these masses remains problematic. If there were persistent malignant disease then immediate surgery or radiotherapy would be indicated. More frequently, the masses consist of necrotic/fibrotic tissue which can be safely observed or, in the case of NSGCT, residual benign teratoma which may be removed as a planned procedure. While anatomical imaging with computerised tomography (CT) clearly identifies residual masses it is unhelpful in determining whether the mass contains residual disease. The measurement of serum tumour markers may be an indicator of persistent disease but unfortunately these are not sensitive or specific enough and cannot indicate the specific site of the disease relapse (Mostofi et al, 1990; Javadpour, 1992).

Functional imaging methods (radiolabelled antibodies, gallium-67 and thallium-201) have been used to determine disease presence (Kalofonos et al, 1990; Uchiyama et al, 1994; Warren, 1995) in patients with testicular tumour. In this study we have explored the use of $^{18}$F fluorodeoxyglucose (FDG) positron emission tomography (PET) to identify active disease by metabolic activity rather than anatomical size. Potentially this approach could: detect small volume disease in solitary residual masses or in lymph nodes that are not enlarged by CT criteria; identify which mass is the site of recurrence when the patient has multiple residual masses and identify additional sites unrelated to the known masses. These techniques could therefore impact on clinical decisions determining the type of intervention required.

*Received 21 January 2000*
*Revised 8 May 2000*
*Accepted 17 May 2000*

*Correspondence to:* SF Hain

### MATERIALS AND METHODS

This study represents a retrospective review of FDG PET scans performed in a series of consecutive patients with germ cell tumours between 1994–1998. All patients were referred by clinical oncologists on clinical grounds. The patient group includes patients with their first relapse and patients who have had a number of relapses and were chemoresistant.

### PET scan protocol

Patients were injected with 320 MBq of $^{18}$F FDG following a 6 hour fast and consecutive 5 minute images from the vertex to mid thigh were obtained on a Siemens ECAT 951 scanner. The images were reconstructed with a spatial resolution of 8 mm FWHM and displayed as projection and coronal, transaxial and sagittal images. Some patients had two position local emission scans over an axial field of view of 10.8 cm each. In these corresponding transmission images were acquired at the start or finish of scanning using 68 Ge rods to enable attenuation correction. All scans were reviewed independently by 2 Nuclear Medicine Physicians and a consensus report was issued.

**Table 1** Patients with residual masses and positive PET scans

| Patients | Numbers of types of tumour NS | S | M | Type of follow up | Biopsy result | FU months | Category |
|---|---|---|---|---|---|---|---|
| 12 | 8 | 4 | – | biopsy | as original histology | 18–66 | TP |
| 5 | 2 | 2 | 1 | clinical response to therapy | – | 22–66 | TP |
| 5 | 3 | 2 | – | clinical progressive disease | – | 18–30 | TP |
| 3 | 3 | – | – | clinical died rapidly from disease | – | <3 | TP |
| 1 | – | 1 | – | microbiology – amoebic abscess | | 36 | FP |

NS = nonseminomatous disease; S = seminoma; M = mixed; TP = true positive; FP = false positive

CT scans were acquired at the participating referring hospitals and the reports issued by the radiologist used to define disease extent. They were reviewed post PET scan and prior to any further treatment.

### Tumour markers

The serum tumour markers βHCG and αFP were measured at the time of the PET scan. The markers measured were βHCG (abnormal results were βHCG > 5 ku/l) and αFP (abnormal results were αFP > 8 u/l).

### Follow-up

Where possible FDG PET results were correlated with histopathology, clinical examination and other imaging modalities to the time of progression, death or to a minimum of 18 months post FDG PET scanning. Without histopathological confirmation, absence of tumour was assumed if there was no progression of the CT abnormalities or that the CT remained or returned to normal and the patient remained well. Active tumour was assumed if there was progression of the known lesion or new lesions identified on conventional imaging during the follow up period.

Mature teratoma differentiated has been regarded as a benign tumour for the purposes of the FDG PET imaging and therefore if FDG PET scans were positive this was regarded as a false positive and if they were negative this was regarded as a true negative.

### RESULTS

Seventy FDG PET scans were performed in 55 men who had separate index events of possible relapse for histologically proven germ cell tumour. The mean age was 30 years (15–55 y). Forty seven scans were performed for assessment of residual masses of which 18 also had raised markers and 23 scans for elevated markers alone with normal or long-term stable disease on CT.

### Residual masses

39 patients (13 seminomas, 24 NSGCT and 2 mixed) had forty-seven FDG PET scans performed to assess residual masses. These patients had either single (23/47) or multiple (24/47) sites of residual masses identified on CT scans.

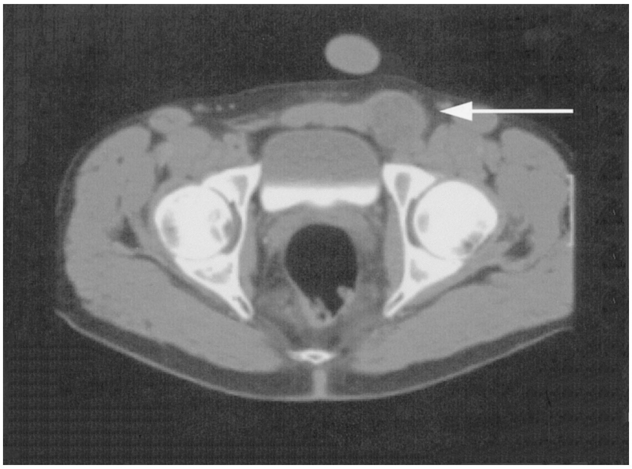

**Figure 1** CT scan of the groin in a patient following chemotherapy and retroperitoneal radiotherapy for seminoma. The CT shows a node in the left groin but is unable to distinguish viable tumour from fibrosis

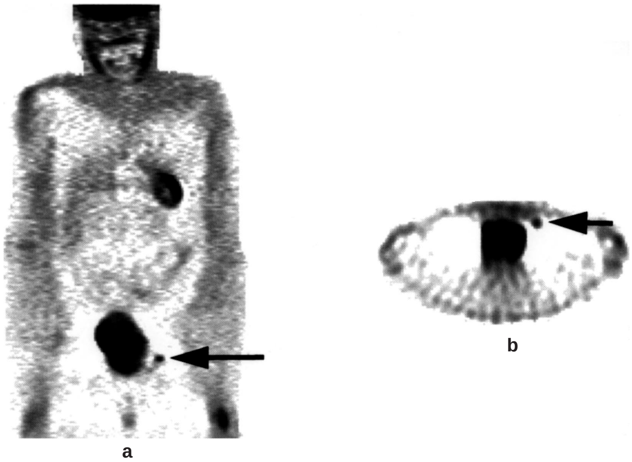

**Figure 2** The PET scan of the patient in Figure 1 clearly showing $^{18}$FDG accumulation in the left groin node. Biopsy showed this mass to contain seminoma

### *FDG PET positive patients*

Twenty-six of the 47 scans were FDG PET positive in one or more sites. Twenty-five of these had convincing evidence of disease

© 2000 Cancer Research Campaign

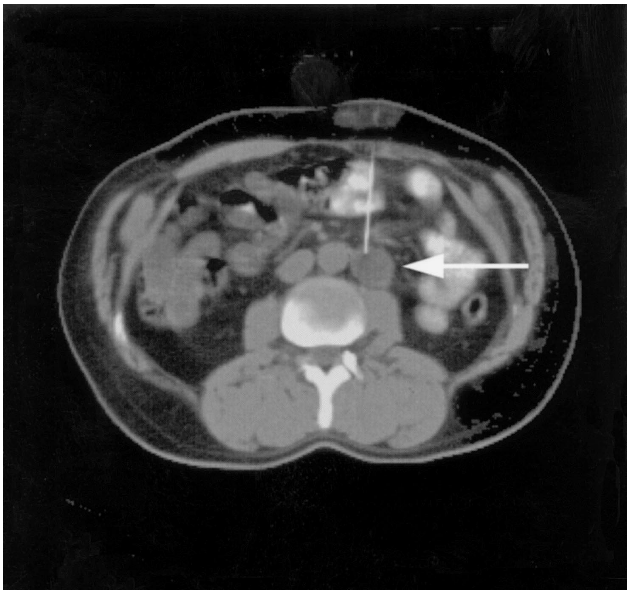

**Figure 3** CT guided biopsy of retroperitoneal lymph node where CT was unable to confirm malignancy. The biopsy was inconclusive

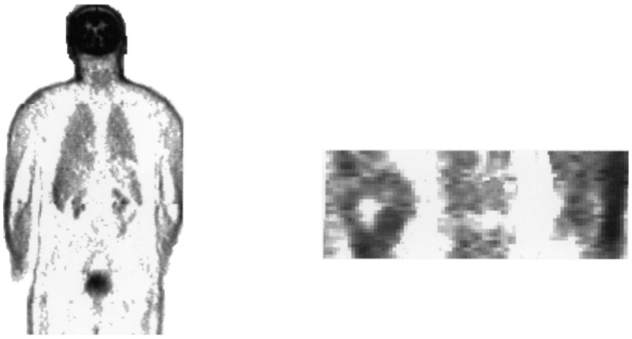

**Figure 4** The PET scan of the patient in Fig. 3 showing no abnormal $^{18}$FDG uptake. Laparotomy showed the node to contain fibrosis only

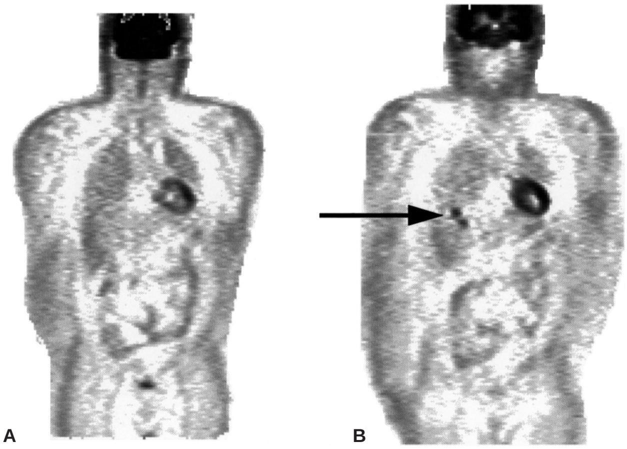

**Figure 5** A patient with raised markers and normal CT had a normal PET scan (**A**). The markers continued to rise, the CT remained normal and the PET scan was the first imaging technology to identify the site of the disease (arrow) (**B**)

(Figs 1 and 2) and there was one false positive scan (Table 1). This was found in a patient who had an amoebic abscess in the liver which was clinically suspected before FDG PET scanning and appropriate antibiotic therapy was given with resolution of the abscess.

### *FDG PET negative patients*

Twenty-one of the 47 scans were negative with eight of these true negative scans (Figs 3 and 4) and 3 false negatives (Table 2). Two patients had very small areas of malignancy in large masses of fibrosis or MTD. The other false negative was in a patient who had undergone PET scanning within 10 days of chemotherapy and this patient had seminoma found on biopsy.

The sensitivity and specificity for FDG PET in residual masses is shown in Table 3.

### Marker positive patients

Forty-one FDG PET scans were performed in patients with elevated markers of which 18 scans were performed in patients with both raised markers and residual masses.

### *FDG PET positive scans*

Twenty-eight of the 41 scans performed were positive (Table 4). Twelve of these were in patients who had raised markers alone and sixteen in patients with residual masses and markers that were raised. Twenty-seven of these scans were true positive scans and one was a false positive in a patient with histologically confirmed MTD.

**Table 2** Patients with residual masses and negative PET scans

| Patients | Type of follow up | Tumour type NS | S | M | Biopsy result | FU months | Category |
|---|---|---|---|---|---|---|---|
| 6 | clinical | 2 | 2 | 2 | none | 18–66 | TN |
| 6 | biopsy | – | 6 | – | negative | 18–73 | TN |
| 6 | biopsy | 4 | 2 | – | non-malignant pathology (MTD in 1) | 18–66 | TN |
| 1 | biopsy | 1 | – | – | MTD + NSGCT | 54 | FN |
| 1 | biopsy | 1 | – | – | fibrosis + NSGCT | 48 | FN |
| 1 | biopsy | – | 1 | – | Seminoma | 54 | FN |

MTD = mature differentiated teratoma; S = seminoma; NS = nonseminomatous germ cell tumour; M = mixed; TN = true negative; FN = false negative

© 2000 Cancer Research Campaign

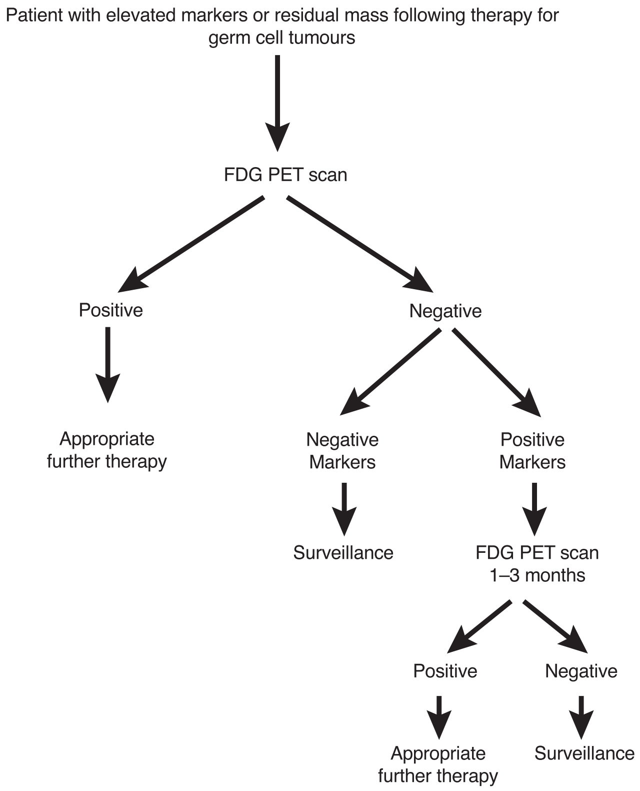

**Figure 6** A algorithm for the use of $^{18}$FDG-PET scanning in patients with residual masses or elevated markers

### *FDG PET negative scans*

Thirteen of forty-one FDG PET scans were negative in patients with elevated markers (Table 5). Seven scans were true negatives and 6 were false negatives. Two patients had a small area of malignant disease, one in a large mass of MTD and one in a fibrotic mass. The other four developed progressive disease within 1–4 months and in three of these PET was the first imaging modality to identify the site and presence of disease (Fig. 5). The 4th patient did not have a subsequent PET scan.

The overall value in terms of sensitivity, specificity and negative and positive predictive value of markers etc. is demonstrated in Table 3.

### Management alteration in all patients

The pre FDG PET plan of management based on the CT findings for the patients were compared with the post PET management which should have occurred with either upgrading or downgrading of disease in the group presenting with residual masses. Twenty-seven (5 seminoma, 20 NSGCT, 2 mixed) of the 47 patients had a change in management as a result of the FDG PET scan. These changes are shown in Table 6.

### DISCUSSION

Traditionally the staging of testicular cancer both at diagnosis and follow up has involved clinical evaluation, CT scanning and marker measurements. It has been recognized that the use of CT for disease assessment is inherently flawed since tumour may be present in 'normal' sized lymph nodes (Saunders et al, 1999) and large masses may contain no tumour. Also tumour markers may be elevated for reasons other than the presence of tumour. These confounding factors following the initial treatment of disease lead to difficulties in deciding whether residual masses contain necrotic tissue, fibrosis, mature teratoma or persistent disease. The latter may need immediate surgical resection but mature differentiated teratoma removal could be delayed and undertaken as a planned procedure with reduction in patient morbidity. Since CT is not able to provide this information for patients with residual masses various functional imaging modalities have been explored and newer techniques such as FDG PET imaging are also being evaluated (de Wit et al, 1997; Bangerter et al, 1998).

There have been few reports of the use of FDG PET in this area. Stephens et al (1996) studied 30 patients with NSGCT with masses post chemotherapy and found that FDG PET was useful to define which patients needed to proceed to surgery. FDG PET did not however distinguish MTD from necrosis or fibrosis, but it did differentiate viable tumour from the other three. This has been confirmed by other studies (Muller-Mattheis et al, 1998) although Sugwara et al (1999) found that the use of kinetic rate constants may differentiate MTD from the other two. Cremerius et al (1998) evaluated 42 post treatment scans (13 within 2 weeks of chemotherapy and 29 more than 2 weeks after chemotherapy) and showed that FDG PET had an accuracy of 90% in determining the presence of active seminoma, providing the scans were performed more than two weeks after chemotherapy. Overall they found FDG

**Table 3** Relative sensitivity, specificity, accuracy and predictive values for PET in the various groups of patients studied (*n* = number of scans)

| | Sensitivity | Specificity | Accuracy | PPV | NPV |
|---|---|---|---|---|---|
| CT with residual masses | | | | 56 | |
| PET with residual masses *n* = 47 | 88 | 95 | 91 | 96 | 90 |
| PET with elevated markers alone *n* = 23 | 71 | 83 | 74 | 92 | 50 |
| PET in all scans *n* = 70 | 81 | 92 | 86 | 95 | 75 |
| markers in all scans *n* = 70 | 76 | 70 | 74 | 80 | 66 |
| markers in scans for residual masses *n* = 47 | 62 | 95 | 76 | 94 | 66 |

*© 2000 Cancer Research Campaign*

**Table 4** Patients with elevated markers and positive PET scans. The patients had either elevated markers alone (CT –ve) or elevated markers with a mass (CT +ve)

| | No of type of tumour | | | | | | | |
|---|---|---|---|---|---|---|---|---|
| Patients | NS | S | M | Type of follow up | CT finding | Biopsy result | FU months | Category |
| 4 | 1 | – | 3 | biopsy | –ve | positive | 18–36 | TP |
| 1 | 1 | | | biopsy | –ve | MTD | 66 | FP |
| 5 | 4 | – | 1 | clinical-response to therapy | –ve | none | 18–24 | TP |
| 1 | 1 | | | clinical-MRI confirmed disease -therapy response | –ve | none | 18 | TP |
| 1 | 1 | | | progressive disease | –ve | none | 18 | TP |
| 16 | 5 | 1 | – | biopsy | +ve | | 18–66 | TP |
| | 3 | 1 | 1 | clinical-response to therapy | +ve | | 22–66 | TP |
| | 4 | 1 | – | clinical-died/progressive disease | +ve | | <3–24 | TP |

NS = non-seminoma; S = seminoma; M = mixed; MTD = mature teratoma; TP = true positive; FP = false positive

**Table 5** Patients with elevated markers and negative PET scans

| | Tumour types | | | | | | | |
|---|---|---|---|---|---|---|---|---|
| Patients | NS | S | M | Type of follow up | CT finding | Biopsy result | FU months | PET category |
| 7 | 4 | 2 | 1 | clinical | –ve | – | 18–66 | TN |
| 2 | 2 | | | biopsy | +ve | small area of teratoma in MTD or fibrosis | 30–36 | FN |
| 4[a] | 2 | 2 | | clinical-progressive disease | –ve | | 18–19 | FN |

MTD = mature differentiated teratoma; NSGCT = nonseminomatous germ cell tumour; TN = true negative; FN = false negative. [a]In three of these patients PET was subsequently positive and was the first imaging modality to demonstrate the site of disease

**Table 6** Management changes based on FDG PET scan findings (XRT = radiotherapy)

| | | Post FDG PET scan therapy | | |
|---|---|---|---|---|
| Pre PET therapy decision | No. | Chemotherapy | Surgery XRT | Clinical Follow-up |
| Chemotherapy | 12 | – | 5 | 7 |
| Surgery/XRT | 15 | 8 | – | 7 |
| Clinical | – | – | – | – |

PET to be superior to CT in the assessment of residual masses and postulated that this could have an effect on patient management. Ganjoo et al (1999) reviewed a smaller number of patients (29 patients) with seminoma in which PET was correctly negative in 19 post initial chemotherapy. In 10 patients who were scanned post salvage chemotherapy 5 patients were correctly negative and 5 relapsed several months later in a PET negative site. None of these 5 patients had PET performed close to the time of relapse and it is difficult to determine from the methods of scanning whether a localized transmission scan was performed over the residual mass. The methods use an arbitrary semiquantitative measure as a cut off for determining disease presence which may also be inaccurate.

The present study shows that FDG PET alone has a good sensitivity and specificity (88 and 95% respectively) for detecting residual disease in masses and also a high negative and positive predictive value (90 and 96%). This compares favourably with the values for sensitivity and specificity of markers in masses (62 and 95%); the negative predictive value of markers however is low at 66%. It is particularly important to note that there were only two false positive scans. One in a patient with a known amoebic abscess which did not provide any source for confusion in the patient management as it is also well known that infective lesions can be visualized with FDG PET scanning (O’Doherty et al, 1997). The other was in a patient with MTD which was surprising since the majority of the MTD lesions were negative on FDG PET. There was no adequate explanation from the histological appearance of the positive finding.

MTD was otherwise found to have no FDG uptake which would be expected since it is essentially a benign condition with a low metabolic rate. These masses do however have the potential to become malignant and are generally removed surgically. The morbidity is higher if the patients are operated on immediately post chemotherapy or radiotherapy especially in those patients with serious comorbid conditions. This finding of a reduced uptake agrees with those of other investigators (Stephens et al, 1996), although Cremerius et al (1998) believed the finding to be a false negative result.

The positive predictive value in masses is extremely high and allows a high degree of certainty with which to decide which patients need further treatment. It may not be felt this detection of malignant residual disease is important in the management of NSGCT; where removal of benign MTD is routinely advised, however, early detection of persistent disease could allow early scheduling of salvage surgery with possible benefits to outcome.

Detection of active disease may be more important in seminoma. Relapses in residual masses occur; but surgical removal is difficult and there is no clear advantage of routine postchemotherapy radiotherapy (Duchesne et al, 1997). Close CT follow up is advised. Use of FDG PET scanning could be helpful

*© 2000 Cancer Research Campaign* 

in this setting to identify patients who could then be treated by radiotherapy.

A negative FDG PET in patients with masses was predictive of the absence of disease, although false negatives did occur. The false negative results were of interest for a number of reasons. One of the false negative results occurred in a patient in whom the scan was performed within 10 days of finishing chemotherapy. Cremerius et al (1998) found a large number of patients who had false negative scans performed within two weeks of chemotherapy. The mechanism for this is uncertain and whether this is a treatment related problem remains to be seen and certainly needs further investigation. It would seem reasonable not to scan within two weeks of chemotherapy in patients with germ cell tumours. The other two false negative results were more concerning since each were due to small volumes of malignancy in large MTD masses. Ganjoo et al (1999) also found 5 patients who relapsed in a residual seminoma mass several months post negative PET scan and the assumption must be that there must have been at least a few malignant cells in the mass at the time of PET scanning. The mass of malignancy detected by FDG PET imaging would be expected to vary depending on the tumour type and metabolic activity. Any imaging procedure is going to have a detection limit but it is possible that the timing of the scan post FDG injection may be an issue in detecting smaller volumes of disease (Lodge et al, 1999). The negative predictive value with standard scanning times is high and is reassuring in most circumstances.

Tumour markers are important in the follow up of patients, and may be the first indicator of relapse (Rathmall et al, 1993). Unfortunately marker negative relapse can occur, even when markers are positive at presentation, also some patients with residual masses can have modest elevations of markers after chemotherapy even though the patients only have necrosis or MTD (Coogan et al, 1997). Similarly a return to normal in elevated markers following treatment does not necessarily correlate with disease remission. (Mostofi et al, 1990; Javadpour, 1992). In tumour marker positive patients there are two areas of difficulty. Firstly when there are multiple residual masses which if any contain malignant disease. Secondly in patients with no residual mass, where if anywhere is the malignancy located and what therapy is required.

In the patients who were marker positive all but one of the PET scans that were positive identified disease. In the group with raised markers alone when FDG PET was positive all scans were true positives 12/12, but when markers were raised in patients with residual masses 15/16 were true positive and only 1/16 false positive suggesting that the combination of a positive FDG PET and positive markers was diagnostic of disease recurrence. FDG PET allowed the identification of the site of disease in these patients. Previous studies using FDG PET could not reach any conclusion with regard to patients with positive tumour markers since most had not had these recorded at the time of scanning (Cremerius et al, 1998). The negative scans were not as predictive of absence of disease with five false negative scans, but in three of these patients a subsequent PET scan was positive and was the only imaging investigation to identify the site of disease. This identification helped in deciding on the optimum management of these patients. Comparing the use of markers and FDG PET scanning overall the FDG PET results showed better results in sensitivity, specificity, accuracy, negative and positive predictive values.

FDG-PET is both sensitive and specific for detecting relapse in patients with germ cell tumour both in patients with raised markers and residual masses. A possible algorithm for investigating patients with germ cell tumours is illustrated in Figure 6. The ability of FDG PET to identify more widespread disease than conventional imaging resulted in a change in management of approximately 57% of patients between local therapy (surgery/radiotherapy) and chemotherapy or surveillance. Although this may appear to be a high percentage of patients with a need for management change, many of these patients had complicated clinical courses and demonstrated chemo-resistance. Thus local treatment is often the only hope of cure and the detection of involved sites becomes very important. In those with first relapse whether there are a few or multiple sites will define the type of consolidation treatment (further chemotherapy or local radiotherapy) post salvage chemotherapy. These changes in management illustrate the potential huge benefits to patients using FDG PET imaging, although a cost effectiveness study needs to be performed. Furthermore the use of FDG PET in the staging of disease at initial presentation needs to be explored since this study demonstrates that these tumours are FDG avid and that this modality was the first to demonstrate disease sites when compared to CT staging.

### REFERENCES

Bangerter M, Moog F, Greisshammer M, Reske SN and Bergman L (1998) Role of whole body FDG-PET in predicting relapse in residual masses after treatment of lymphoma. *Br J Haematol* **102**: 148

Coogan CL, Foster RS, Rowland RG, Bihrle R, Smith ER Jr, Einhorn LH, Roth BJ and Donohue JP (1997) Postchemotherapy retroperitoneal lymph node dissection is effective therapy in selected patients with elevated tumor markers after primary chemotherapy alone. *Urology* **50**: 957–962

Cremerius U, Effert PJ, Adam G, Sabri O, Zimmy M, Wagenkneckt GG, Jaske G and Buell U (1998) FDG PET for detection and therapy control of metastatic germ cell tumour. *J Nucl Med* **39**: 815–822

de Wit M, Bunmann D, Beyer W, Herbst K, Clausen M and Hossfeld DK (1997) Whole body positron emission tomography (PET) for diagnosis of residual mass in patients with lymphoma. *Ann Oncol* (suppl 1) **8**: 57–60

Duchesne GM, Stenning SP, Aass N, Mead GM, Fossa SD and Oliver RTD (1997) Minimal benefit from radiotherapy after chemotherapy for metastatic seminoma: analysis of medical research council (MRC-UK) database. *Proc ASCO* **16**: 318

Ganjoo KN, Chan RJ, Sharma M and Einhorn LH (1999) Positron emission tomography scans in the evaluation of postchemotherapy residual masses in patients with seminoma. *JCO* **17**: 3457–3460

Javadpour N (1992) Current status of tumor markers in testicular cancer. A practical review. *Eur Urol* **21**: 34–36

Kalofonos HP, Kosmas C, Pawlikowska TR, Bamias A. Snook D. Dhokia B. Sivolapenko GB and Courtenay-Luck NS (1990) Immunolocalisation of testicular tumours using radiolabelled monoclonal anitbody to placental alkaline phosphatase. *J Nucl Med Allied Sci* **34**: 294–298

Lodge MA, Lucas JD, Marsden PK, Cronin BF, O'Doherty MJ and Smith MA (1999) A PET study of $^{18}$FDG uptake in soft tissue masses. *Eur J Nucl Med* **26**: 22–30

Mead GM (1995) Testis. In: *Treatment of Cancer*. 3rd edn, Price P and Sikora K (eds) pp 627–645. Chapman Hall, London

Mostofi FK, Spaander P, Grigor K, Parkinson CM, Skakkekaek NE and Oliver RTD Consensus on pathological classifications of testicular tumours (1990) In: *EORTC genitourinary group monograph 7. Prostate cancer and testicular cancer*, pp 267–27; Wiley-Liss, New York

Muller-Mattheis V, Reinhardt M, Gerharz CD, Feurst G, Vosberg H, Meullwe-geartner HW and Ackermann R (1998) Positron emission tomography with [18F]-2-fluoro-2-deoxy-D-glucose in the diagnosis of retroperitoneal lymph node metastases of testicular tumors. *Urologe-Ausgabe* A. **37**: 609–620

O'Doherty MJ, Barrington SF, Campbell M, Owen J and Barber CS (1997) PET scanning and the human immunodeficiency virus positive patient. *J Nucl Med* **38**: 1575–1583

Rathmall AJ, Brand IR, Carey BM and Jones WG (1993) Early detection of relapse after treatment for metastatic germ cell tumour of the testis: an exercise in medical audit. *Clin Oncol R Coll Radiol* **5**: 34–38

© 2000 Cancer Research Campaign

Saunders CAB, Dussek J, O'Doherty MJ and Maisey MN (1999) An evaluation of 18F-FDG whole body PET imaging in the staging of lung cancer. *Ann Thoracic Surg* **67**: 790–797

Stephens AW, Gonin R, Hutchins GD and Einhorn LH (1996) Positron emission tomography of residual radiological abnormalities in postchemotherapy germ cell tumour patients. *J Clin Oncol* **14**: 1637–1641

Sugawara Y, Zasadny KR, Grossman HB, Francis IR, Clarke MF and Wahl RL (1999) Germ cell tumour: differentiation of viable tumor, mature teratoma and necrotic tissue with FDG PET and kinetic modelling. *Radiology* **211**: 249–256

Uchiyama M, Kantoff PW and Kaplan WD (1994) Gallium-67 citrate imaging extragonadal and gonadal seminomas: relationship to radiologic findings. *J Nucl Med* **35**: 1624–1630

Warren GP (1995) Gallium scans in the evaluation of residual masses after chemotherapy for seminoma. *J Clin Oncol* **13**: 2784–2788

*© 2000 Cancer Research Campaign*
